# V3 Interneurons Are Active and Recruit Spinal Motor Neurons during *In Vivo* Fictive Swimming in Larval Zebrafish

**DOI:** 10.1523/ENEURO.0476-21.2022

**Published:** 2022-03-28

**Authors:** Timothy D. Wiggin, Jacob E. Montgomery, Amanda J. Brunick, Jack H. Peck, Mark A. Masino

**Affiliations:** 1 Department of Neuroscience, University of Minnesota, Minneapolis, MN 55455; 2 Neuroscience Program, Bucknell University, Lewisburg, PA 17837

**Keywords:** locomotion, motor neuron recruitment, spinal interneuron, zebrafish

## Abstract

Survival for vertebrate animals is dependent on the ability to successfully find food, locate a mate, and avoid predation. Each of these behaviors requires motor control, which is set by a combination of kinematic properties. For example, the frequency and amplitude of motor output combine in a multiplicative manner to determine features of locomotion such as distance traveled, speed, force (thrust), and vigor. Although there is a good understanding of how different populations of excitatory spinal interneurons establish locomotor frequency, there is a less thorough mechanistic understanding for how locomotor amplitude is established. Recent evidence indicates that locomotor amplitude is regulated in part by a subset of functionally and morphologically distinct V2a excitatory spinal interneurons (Type II, nonbursting) in larval and adult zebrafish. Here, we provide direct evidence that most V3 interneurons (V3-INs), which are a developmentally and genetically defined population of ventromedial glutamatergic spinal neurons, are active during fictive swimming. We also show that elimination of the spinal V3-IN population reduces the proportion of active motor neurons (MNs) during fictive swimming but does not alter the range of locomotor frequencies produced. These data are consistent with V3-INs providing excitatory drive to spinal MNs during swimming in larval zebrafish and may contribute to the production of locomotor amplitude independently of locomotor frequency.

## Significance Statement

Currently, there is a limited understanding about the cellular and spinal network properties that produce locomotor amplitude, defined as limb displacement in limbed animals or tail-bend in nonlimbed animals during locomotion. Here, we show, directly for the first time in a vertebrate, that V3 interneurons (V3-INs) in zebrafish larvae are active during *in vivo* fictive locomotion, and that targeted ablation of the spinal V3-IN population reduces the proportion of active motoneurons during fictive swimming. Importantly, ablation of V3-INs does not affect locomotor frequency (speed), which clarifies their role in motor control rather than rhythm generation. Thus, we propose that the V3-IN population is a source of excitation in the vertebrate locomotor neural circuitry that may participate in regulating locomotor amplitude.

## Introduction

Efficient locomotion is set by a combination of kinematic properties, including the frequency and amplitude of rhythmic motor output. These properties combine to determine various locomotor features, such as distance traveled, speed, and vigor (Webb, 1975; Jayne and Lauder, 1995; Donley and Dickson, 2000; Tytell, 2004). Locomotor frequency (e.g., limb or tail-beat rhythmic activity) is a well-studied kinematic variable, which has been shown to increase linearly with speed (Bainbridge, 1958; Hunter and Zweifel, 1971; Webb et al., 1984; Müller and van Leeuwen, 2004). Less well studied is locomotor amplitude, which is measured by limb displacement in limbed animals during locomotor events (walking, trotting, galloping, hopping; Heglund et al., 1974) or by tail-bend in nonlimbed animals during anguilliform swimming (Wardle et al., 1995; Videler et al., 1999). Although there is a fundamental understanding of how identified subsets of excitatory spinal interneurons help to establish the frequency of motor output (Ausborn et al., 2012; Eklöf-Ljunggren et al., 2012; Kimura et al., 2013; Ampatzis et al., 2014; Ljunggren et al., 2014; Menelaou et al., 2014), a more complete understanding of the cellular and network properties that regulate locomotor amplitude in vertebrate animals is lacking.

Across vertebrate species from fish to mammals, the neural circuitry that controls locomotion is located in the ventromedial spinal cord (Grillner and Jessell, 2009; Kiehn, 2011). In vertebrates, developmental gene expression specifies at least five cardinal classes of ventral neuronal progenitor cells. These progenitors give rise to motor neurons (MNs) and four classes of ventral interneurons, referred to as V0 to V3 neurons (Ericson et al., 1997; Pierani et al., 1999). Over the past decade, a population of identified excitatory spinal interneurons (V2a) has been shown to control the speed of locomotion in larval and juvenile/adult zebrafish (Ausborn et al., 2012; Ampatzis et al., 2014). More recent studies indicate a role for a subset of V2a interneurons (Type II, nonbursting) in regulating locomotor amplitude (Menelaou and McLean, 2019) and/or vigor (Song et al., 2018) in larval and adult zebrafish, respectively. Both studies reveal a hierarchical control of MN recruitment by a diverse V2a interneuron population, which the authors conclude is the basis for regulating locomotor amplitude and/or vigor.

A cardinal class of spinal interneurons in the ventral cord that selectively regulates locomotor amplitude has been elusive. In the mammalian spinal cord, a genetically defined population of premotor glutamatergic neurons, called V3 interneurons (V3-INs), contributes to limb coordination, motor burst duration, and muscle spasms, but does not have a clear role in locomotor pattern generation (Chopek et al., 2018; Danner et al., 2019; Lin et al., 2019). Recently, neurons derived from the p3 progenitor domain were shown to modulate locomotor adaptation in larval zebrafish (Böhm et al., 2022). We hypothesized V3-INs provide excitatory drive during locomotor activity to regulate locomotor frequency, amplitude, or both. The work presented here shows directly that V3-INs in zebrafish larvae are active during *in vivo* fictive locomotion (swim-active), and that elimination of the V3-IN spinal population reduces the proportion of active MNs during fictive swimming but does not alter the range of locomotor frequencies produced. These data are consistent with V3-INs providing excitatory drive to spinal MNs during swimming in larval zebrafish and may contribute to the production of locomotor amplitude independently of locomotor frequency. This is conceptually important because it clarifies a role of the V3 cardinal class of spinal interneurons in motor control rather than rhythm generation.

## Materials and Methods

### Fish lines and maintenance

All animal procedures were performed in accordance with the animal care committee’s regulations. Wild-type (Segrest Farms) and transgenic adult zebrafish (*Danio rerio*) were maintained in the animal facility. The transgenic lines used in these experiments were: *Tg(nkx2.2a:mEGFP)^vu17^* (Kirby et al., 2006), *Tg(vglut2a:DsRed)^nns9^* (Miyasaka et al., 2009), *Tg(vglut2a:Gal4ff)^nns20^* (Satou et al., 2013), *Tg(UAS:GCaMP6s)^nk13a^* (Muto et al., 2017), and *parga^mn2Et^* (Balciunas et al., 2004). Out-crossed transgenic larvae at 4–6 d postfertilization (dpf) were used in these experiments. Larval zebrafish were maintained in Petri dishes filled with embryo water (60 μg/ml Instant Ocean salt mix) and 0.00005% Methylene Blue in a 28.5°C incubator with a 14/10 h light/dark cycle (Zeitgeber time 0 = 8:30 A.M.).

### V3-IN soma distribution analysis

Quantification of the distribution of V3-IN cell bodies was performed using Fiji and custom MATLAB code (MathWorks). A confocal microscope (Olympus, FV-100) was used to collect overlapping stacks of images documenting the expression of *Tg(vglut2a:DsRed)^nns9^* and transmitted light morphology. Images were acquired at high magnification (40×/NA 0.8 objective) and a *z-axis* resolution of 1.3 μm. The fields of view in each stack overlapped with their neighbors by at least 10% of the width to permit accurate stitching and included the full width of the spinal cord and dorsal root ganglia (DRG) in the field of view. The collection of stacks was stitched into a single 3-D image of the caudal hindbrain and spinal cord (Preibisch et al., 2009). The Fiji Cell Counter tool (Schindelin et al., 2012) was used to identify the coordinates of all V3-INs in the rostral 2 mm of the larval spinal cord (out of a total length of ∼3 mm), the coordinates of the DRG neuron clusters, and the position of the dorsal and ventral edges of the spinal cord. The markers tracing the dorsal and ventral edges of the spinal cord were used to calculate local tangent lines to the dorsal and ventral edges of the spinal cord at the rostrocaudal position of each V3-IN. The equation of a line perpendicular to the ventral tangent and intersecting the V3-IN was calculated, referred to as the “V3 plumb line.” The rostrocaudal distance of each V3-IN was calculated as the distance along the ventral surface of the spinal cord between the spinal cord/hindbrain junction [segment (s)2/3 boundary] and the V3 plumb line intersection with the ventral surface. The dorsoventral height of the spinal cord at the location of each V3-IN was calculated as the distance between the intersections of the V3 plumb line with the dorsal surface and the ventral surface, respectively. The distance of the V3-IN from the ventral surface of the spinal cord was also measured along the plumb line. The *z*-axis position of DRG neurons on each side of the spinal cord were used as boundary points of the lateral edges of the spinal cord, and a linear fit to the DRG neurons on each side of the animal was used to calculate the edges of the spinal cord at each rostrocaudal position in the imaging area. The midline slice and spinal cord width were calculated at the rostrocaudal position of each V3-IN, along with the distance from midline of the V3-IN.

### Kaede photoconversion

*In vivo* photoconversion of genetically encoded Kaede was performed in *Tg(vglut2a:Gal4ff)^nns20^;Tg(UAS:Kaede)^s1999t^* larvae using a fixed-objective, upright microscope (Olympus FV-1000, 60×/NA 1.0 objective). Larvae were anesthetized in zebrafish Ringer’s solution (Wiggin et al., 2012, 2014) with 0.02% tricaine-S (MS-222; Western Chemical), embedded laterally in a fluorodish (World Precision Instruments) filled with 1.5% low-melting point agarose in zebrafish Ringer’s solution and covered with additional saline containing anesthetic. Photoconversion of Kaede was performed using the bleach function of the FV-1000 to direct a 405-μm laser spot onto a Kaede-expressing cell targeted for conversion (5% laser intensity, 7 s, single pulse). The embedding procedure resulted in small variations in the orientation of the spinal cord such that the larvae were not precisely level and square with respect to the imaging plane. Following confocal stack acquisition, Fiji (Schindelin et al., 2012) was used to rotate the image to correct these errors.

### Whole-mount immunohistochemistry

Five dpf *parga^mn2Et^* larvae were killed with 0.2% tricaine-S and fixed overnight in 4% paraformaldehyde at 4°C. Whole-mount larvae were permeabilized with Proteinase K, postfixed, and blocked with 0.2% BSA, 10% normal donkey serum, 0.5% Triton X-100 in PBS. Goat polyclonal anti-choline acetyltransferase (ChAT; 1:50; AB144P) was added to the blocking solution and incubated overnight at 4°C. Fixed larvae were incubated with Alexa Fluor 633 donkey anti-goat secondary antibody (1:500; A-21 082, Thermo Fisher Scientific Inc.) in secondary blocking solution (0.2% BSA, 1% normal donkey serum, 0.5% Triton X-100 in PBS) for three nights at 4°C.

Anti-ChAT-labeled *parga^mn2Et^* larvae were embedded laterally in low melting-point agarose. An Olympus Fluoview FV1000 confocal microscope was used to collect images through a 60× objective centered on body S15. Confocal stacks contained the entire dorsoventral extent of the spinal cord and spanned approximately three body segments (285 μm). Images were processed in Fiji and labeled cells from a single side of the spinal cord (approximately three hemisegments) were quantified using the Cell Counter plugin. Photoshop CS5 (Adobe Systems) was used to adjust brightness and contrast across entire z-projected images.

### *In vivo* Rhod-2 AM dye loading of MNs

For *in vivo* characterization of MNs in *parga^mn2Et^* larvae, a 0.25% Rhod-2 AM (Life Technologies) stock was made by dissolving 50 μg of dye in 20-μl Pluronic F-127:DMSO (1:4; Life Technologies). Next, the skin was removed from the midbody region and a 1:50 dilution of the Rhod-2 AM stock was bath applied to the exposed muscles to permit sequestration of indicator dye by MNs via the peripheral motor axons. Sensory neurons were not strongly loaded by this procedure. Following 1-h incubation, the Rhod-2 AM solution was thoroughly washed-out using zebrafish Ringer’s solution. Confocal images were collected, and cells were quantified as described for whole-mount immunohistochemistry (above).

### Peripheral nerve (PN) recordings and analysis

Zebrafish larvae were anesthetized, the skin was carefully removed from the recording region, and larvae were paralyzed with 0.1 mm α-bungarotoxin (Tocris) to prevent muscle contractions. The anesthetic was removed to allow the larvae to perform fictive behavior, and a suction electrode (9–15-μm tip diameter) was placed over the intermyotomal cleft to record MN axons. Fictive motor bursts were identified and grouped into episodes using custom MATLAB code. The program detected the presence or absence of activity at each voltage sample v(n). For each v(n), the algorithm determined a voltage autocorrelation $c_{n}\left(k\right)$ over a small window (3 ms) centered at v(n). These “windowed” autocorrelations were computed as:

(1) $$c_{n}\left(k\right)=\sum_{i}{{}_{=-N0}}^{i}{{}^{=N}}_{0}v\left(n-i\right)v\left(n–i-k\right)$$

where 3-ms windowing was implemented in Equation 1 by setting $N_{0}=\left(\text{3 ms}\cdot f_{\text{sam}}\right)/\text{2}$, where $f_{\text{sam}}$ is the sampling frequency, and by setting v(j)=0 for *j* outside the interval $\left[n-N_{0},n+N_{0}\right]$.

A subset of the autocorrelation values (lags) from Equation 1 were used to compute a test statistic for each v(n) with the same lags $\left(k\to_{0}=\left[k_{\text{1}},k_{\text{2}},...,k_{m}\right]\right)$ used for all voltage samples. Building on Equation 1, for each v(n), a test statistic $c_{n}$ was computed as:

(2) $$c_{n}=\sum_{k}{{}_{=k\text{1}}}^{k}{{}^{=k}}_{m}\sum_{i}{{}_{=-N0}}^{i}{{}^{=N}}_{0}v\left(n-i\right)v(n-i-k)$$

where Equation 2 is the sum of the $c_{n}\left(k\right)$ from Equation 1 specified by $k\to_{0}.k\to_{0}$ was set at $k\to_{0}=\left[\text{1},\text{ 2}\right]$ because we found that these values effectively separate the distributions of the test statistics $\left\{c_{n}\right\}$ for samples of noise and samples of activity across a broad range of recording quality.

Finally, activity was considered present at v(n) only when $c_{n}$ was greater than a detection threshold *T. T* was set as the maximum of a set of $\left\{c_{n}\right\}$ corresponding to the {v(n)} in one contiguous second of the voltage recording where activity was confirmed to be absent (typically the first second of the recording), and was set this way for each individual voltage recording to account for differences in baseline noise levels. Fictive locomotor bursts were detected and grouped into episodes, and the burst and episode properties were determined as follows. Episode duration is the time from the onset of the first burst of an episode to the offset of the final burst in the same episode. Burst duration is the time from the onset to the offset of each burst, as defined by *c_n_* and described above. Burst frequency is the inverse of the interburst period (IBP), which is defined for each pair of bursts within episodes as the time from the onset of the first burst to the onset of the second burst. Peak burst frequency is the inverse of burst period, which is defined as the shortest time duration from onset of one burst to onset of the subsequent burst in an episode of fictive swimming. IBPs between episodes are excluded from burst frequency analysis.

### Calcium imaging and analysis

For calcium imaging of V3-INs, *Tg(vglut2a:Gal4ff)^nns20^;Tg(UAS:GCaMP6s)^nk13a^* zebrafish larvae were used. For calcium imaging of MNs, Calcium Green-1 AM (Life Technologies) dye was loaded into MNs using the protocol for Rhod-2 AM dye loading described above. Larvae were transferred to a fixed-stage Olympus BX51 WI upright microscope with either a 20×/NA 0.5 or a 40×/NA 0.8 water dipping objective lens. A field of view containing *GCaMP6s*-expressing V3-INs in transgenic larvae or Calcium Green-1 AM-loaded MNs in wild-type larvae was selected for calcium imaging and a simultaneous PN recording was acquired. Calcium indicator fluorescence was excited with an E-Cite Series 120 Q lamp (Excelitas Technologies) and acquired at 20 Hz with a Retiga EXi camera and QCapture Pro 6.0 software (QImaging). Data were acquired during spontaneous fictive swimming in 10- to 60-s durations for both V3-IN and MN trials. Multiple trials (typically 4) were acquired for each field of view, with at least 5 min between trials. Movement artifacts during data acquisition were corrected in Fiji by registering frames from all trials to a common reference image (Preibisch et al., 2009). Optical and electrophysiological data acquisition were synchronized by using the sync output of the stimulator to trigger acquisition of the PN recording.

For all calcium imaging experiments, raw fluorescence values were determined by drawing a region of interest around each identified V3-IN or MN soma and using the Plot *z*-axis Profile command in Fiji. Fluorescence from a region of interest containing background signal was subtracted from raw soma fluorescence measurements to reduce the effect of photobleaching. Baseline fluorescence (F_0_) was defined as the mean fluorescence in the first 10 frames of the recording. To calculate ΔF/F, F_0_ was subtracted from fluorescence for each frame [F_(t)_], then divided by F_0_; ΔF/F = (F_(t)_ – F_(0)_)/F_(0)_. A neuron was considered active if ΔF/F was 10% or greater.

### Targeted laser ablation of V3-INs

Acute ablation of the V3-INs was performed using a Micropoint pulsed nitrogen pumped dye laser (Andor Technology). Light (λ = 435 nm) was delivered via an Olympus BX51 WI microscope using a 60×/NA 1.0 water dipping objective lens. The laser was focused to apply maximal light intensity to the imaging plane of the microscope, the intensity of the laser was calibrated to produce a 4.5- to 5.0-μm hole in a mirror slide (this optimal intensity was determined empirically), and the x-y coordinates of the laser point in the field of view were indicated on a digital display. Three to 6 dpf *Tg(vglut2a:DsRed)^nns9^* zebrafish larvae were anesthetized in 0.02% tricaine in zebrafish Ringer’s solution and embedded laterally in a gel composed of 3% methylcellulose and 0.02% tricaine in zebrafish Ringer’s saline. A coverslip was placed over the gel and secured at the edges with agarose to stabilize the position of the larvae.

Targeted, bilateral laser ablation of DsRed^+^ V3-INs in the ventral spinal cord was performed by positioning the microscope objective such that a neuron was in the *x-y* position at which the laser was focused. Laser pulses were applied to each V3-IN soma at 33 Hz for 10 s. Laser intensity was empirically determined a priori such that >95% of the targeted V3-INs were not present 24 h postablation. Following the train of laser pulses, the microscope was repositioned to focus on another identified V3-IN, and the protocol was repeated. Larvae were embedded for ∼50 min to perform bilateral ablation of ∼140 identified V3-INs between body S5 and S20. To confirm that laser ablation of targeted V3-INs was effective and persistent, the lack of DsRed fluorescence in the lesioned spinal cord of *Tg(vglut2a:DsRed)^nns9^* larvae was confirmed ∼24 h postablation. Sham larvae were treated identically to V3 ablation larvae, but the x-y position of the laser was offset by 5-20 μm from each V3 cell, producing nonspecific damage to neurons other than the V3 population. Rather than kill a specific cell type as a sham control which might have a specific effect, nonspecific laser damage was preferred.

### Statistical analysis

Statistical analysis of data were performed using two-sample *t* test, ANOVA test, Holm–Sidak *post hoc* test, Kolmogorov–Smirnov test, and least-squares linear regression. Statistical tests were conducted using SigmaPlot 12 and 14 software (SyStat Software), MATLAB (MathWorks), or Microsoft Excel (Microsoft). An α level of 0.05 was used to determine statistical significance. Data are reported as means and SD.

### Software accesibility

Custom MATLAB code for the quantification of the distribution of V3-IN cell bodies and PN recording analysis is available on request.

## Results

### V3-INs are individually identifiable in the larval zebrafish spinal cord

To characterize the functional roles of V3-INs *in vivo*, it was necessary to reliably target the neurons for recording and perturbation. While there are no published transgenic zebrafish lines that specifically label neurons expressing *sim1a* (the zebrafish V3-IN marker; Schäfer et al., 2007), it is possible to infer V3-IN identity from their other characteristics. V3-INs proliferate ventral to the central canal and are glutamatergic (Zhang et al., 2008; Yang et al., 2010). In zebrafish, there is >90% overlap of *vesicular glutamate transporter 2* (*vglut2a*) and *sim1a* expression in the ventral spinal cord at the embryonic stage (1.5 dpf; Yang et al., 2010). However, it is possible that the embryonic ventral glutamatergic neurons migrate or are replaced by a non-V3 population between 1.5 dpf and the free-swimming larval stage (>3 dpf).

**Figure 1.**
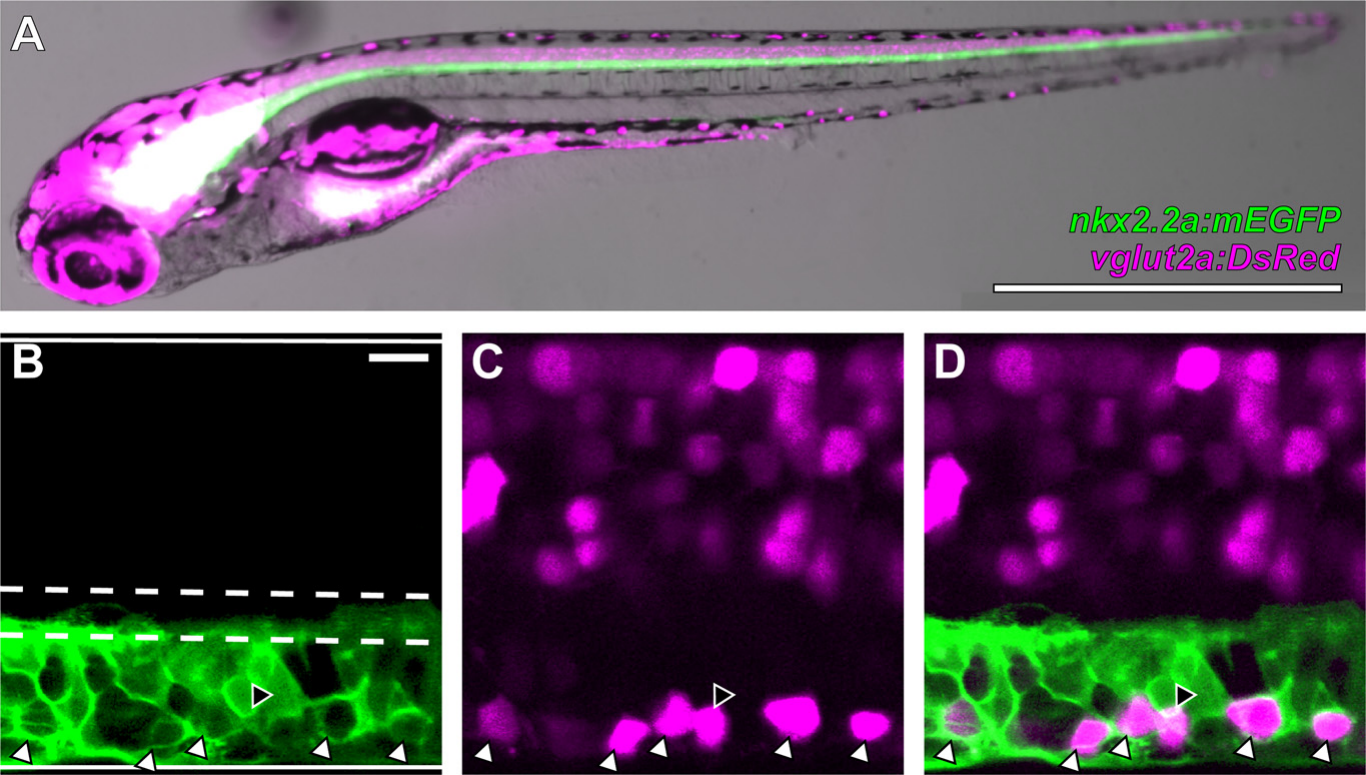
V3-INs are identified by co-localization of *vglut2a:DsRed* and *nkx2.2a:mEGFP* transgene expression in neurons located in the ventromedial spinal cord. ***A***, Whole-mount *Tg(nkx2.2a:mEGFP)^vu17^*;*Tg(vglut2a:DsRed)^nns9^* double transgenic zebrafish larva. ***B–D***, A representative single confocal optical section of the spinal cord exhibiting: (***B***) *nkx2.2a:mEGFP* expression, (***C***) *vglut2a:DsRed* expression, and (***D***) overlayed *nkx2.2a:mEGFP* and *vglut2a:DsRed* expression. The boundaries of the spinal cord and central canal are illustrated by solid and dashed white lines, respectively, in panel ***B***. White triangles indicate neurons that co-express both transgenes and the black triangle indicates the only *vglut2a:DsRed* neuron ventral to the central canal that did not co-express *nkx2.2a:mEGFP* in the field of view. Scale bars: 1 mm (***A***) and 10 μm (***B–D***).

To test the hypothesis that the ventral *vglut2a:DsRed*-expressing neurons observed in 4–6 dpf *Tg(vglut2a:DsRed)^nns9^* larvae are V3-INs, we crossed this line to *Tg(nkx2.2a:mEGFP)^vu17^*, which marks p3 progenitor neurons (Briscoe et al., 2000; Schäfer et al., 2007; Fig. 1*A*). At 4–6 dpf, *nkx2.2a:mEGFP* expression was restricted to the region ventral to the central canal (Fig. 1*B*). The p3 domain of the zebrafish spinal cord also produces Kolmer–Agduhr neurons (Yang et al., 2010), and likely other neuronal types, so it was expected that there would be more *nkx2.2a:mEGFP*-expressing neurons than *vglut2a:DsRed*-expressing neurons (Fig. 1*C*). Approximately 94% of *vglut2a:DsRed*-expressing neurons ventral to the central canal also expressed *nkx2.2a:mEGFP* (*n *=* *6 larvae, 162 neurons), and were therefore confirmed to be V3-INs (Fig. 1*D*). There was not a significant difference between the dorsoventral position of the *vglut2a:DsRed*-expressing neurons that co-expressed *nkx2.2a:mEGFP* and those that did not (two-sample *t* test; *t *=* *1.6; *p *=* *0.10), so there was not a way to reliably segregate the identified V3-INs, from those that did not express *nkx2.2a:mEGFP*. However, the overwhelming majority of the DsRed^+^ neurons ventral to the central canal were confirmed to be V3-INs. The lack of confirmed transgene overlap in the remaining cells may be because of variegated transgene expression rather than the presence of a non-V3-IN ventral glutamatergic neuronal population. Based on this experiment, we treat the ventral *vglut2a:DsRed*-expressing neurons as the V3-IN population, with the caveat that a small fraction (∼6%) may be misidentified.

### V3-INs are located in the ventromedial spinal cord and are distributed along the rostrocaudal axis of the spinal cord

**Figure 2.**
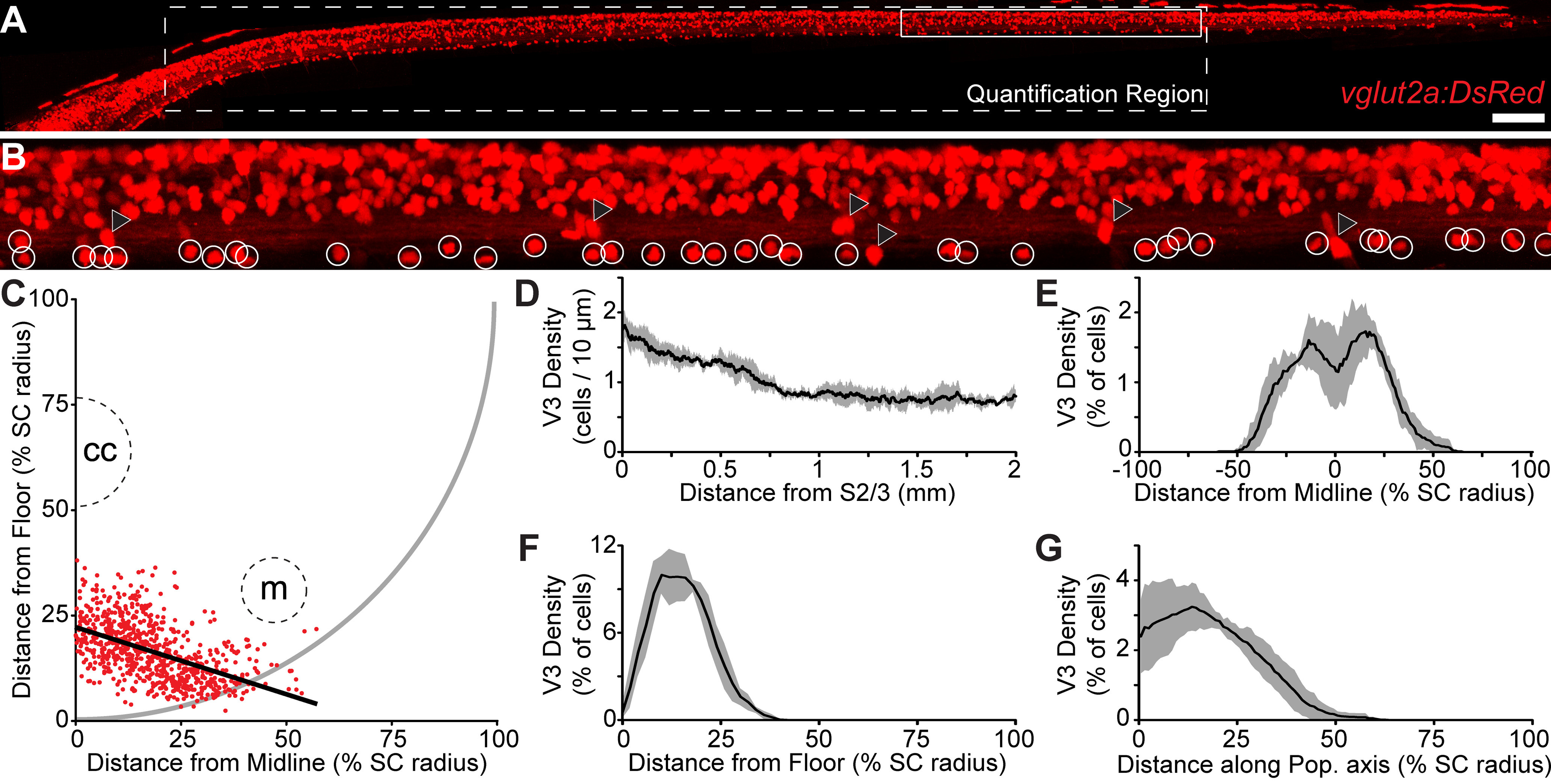
Distribution of V3-IN cell bodies in the spinal cord. ***A***, Maximum intensity projection of a confocal stack of the spinal cord of a 5 dpf Tg(*vglut2a:DsRed*)*^nns9^* zebrafish larva. The dashed white outline indicates the region of the cord in which cells were quantified. The solid white outline is the region of the image shown at higher magnification in ***B***. ***B***, A higher magnification field of view. White circles indicate V3-INs, black triangles indicate dorsal root ganglion neurons located lateral to the spinal cord. ***C***, Distribution of all quantified V3-INs (red dots) projected in the transverse plane of the spinal cord. The schematic outline of the spinal cord (solid gray line), as well as the location of the central canal (cc) and Mauthner axon (m), are approximate. The heavy black line indicates the line of best fit that best describes the axis of the V3-IN population. ***D–G***, The windowed moving average of V3-IN density along the rostrocaudal axis (***D***; window width: 250 μm), mediolateral axis (***E***; window width: 15% SC radius), dorsoventral axis (***F***; window width: 15% SC radius), and along the population axis (***G***; window width: 15% SC radius). Black lines are the mean, gray area indicates the SD. SC: spinal cord. Scale bar: 100 μm (***A***) and 19 μm (***B***).

In mice, the V3-INs migrate following differentiation to form spatially segregated dorsal and ventral populations that have different cellular properties and may be necessary for different behaviors (Borowska et al., 2013). To determine whether there are multiple populations of spatially segregated V3-INs in zebrafish larvae, we used confocal microscopy to create 3-D reconstructions of the spinal cords of 4–6 dpf *Tg(vglut2a:DsRed)^nns9^* zebrafish larvae (Fig. 2*A*,*B*). The center of each V3-IN soma in the most rostral 2 mm of the spinal cord was tagged, and the distribution of the neurons within the spinal cord was calculated based on the imputed borders of the spinal cord (see Materials and Methods). Neurons in the far caudal spinal cord were not counted because the dimensions of the spinal cord taper to the extent that it was difficult to determine the location of the central canal and Mauthner axons. All V3-INs (*n *=* *4 larvae; 797 neurons) were located medial of the Mauthner axon (Fig. 2*C*). V3-IN density was highest in the rostral spinal cord but remained close to 1 cell per 10 μm throughout the cord (Fig. 2*D*). The V3-IN somas were ∼5 μm in diameter in the rostrocaudal axis, so a density of ∼1 cell per 10 μm thoroughly tiled the ventral spinal cord. The majority of V3-INs were located in the most medial and most ventral 25% of the spinal cord (Fig. 2*E*,*F*). There was no parasagittal plane through the medial spinal cord that was unoccupied by V3-INs (Fig. 2*E*), which suggested that the V3-IN population may be considered a midline population. However, the density of V3-INs was not highest at the sagittal midline of the spinal cord, and when cell density was plotted along the population axis, the point of highest density was adjacent to the spinal cord midline (Fig. 2*G*). Therefore, we propose that distinct left and right populations of V3-INs are present, but that there are not multiple ipsilateral populations of spatially segregated V3-INs in larval zebrafish.

### V3-INs project bilaterally and have descending processes

**Figure 3.**
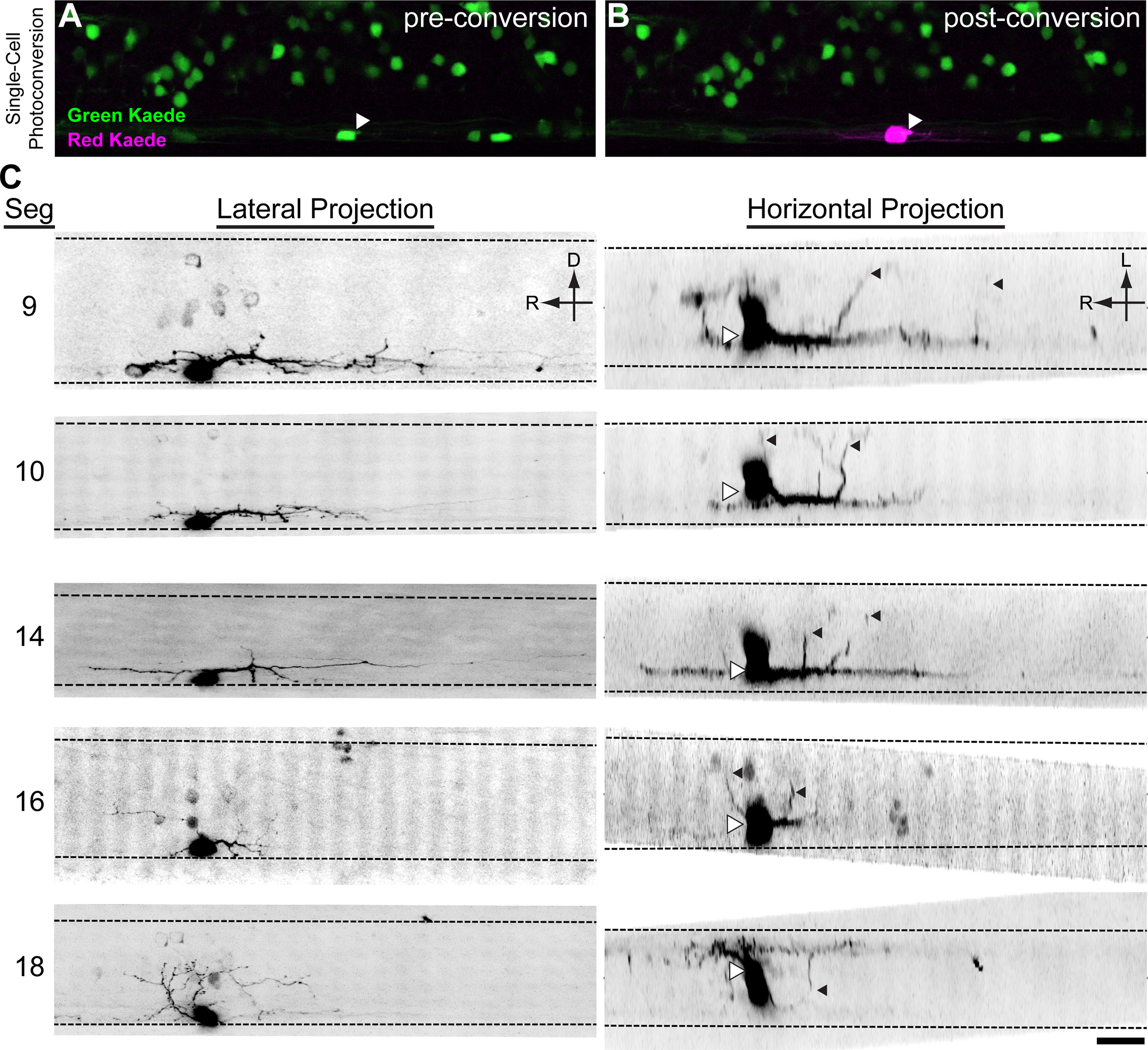
V3-INs are descending, bilaterally projecting cells. Kaede was expressed in *vglut2a*-expressing neurons in *Tg(vglut2a:Gal4ff)^nns20^*;*Tg(UAS:Kaede)^s1999t^* double transgenic larvae (***A***). A single V3-IN was exposed to UV laser light, converting the Kaede protein from green fluorescence to red (***B***). ***C***, Inverted contrast projections of Kaede-red fluorescence in five neurons. Each row of images is a neuron shown in a lateral z-projection (left) and horizontal z-projection (right). On the horizontal projections, the center of the cell body is indicated with white triangles to account for optical bloom and contralateral projections are indicated with black triangles. Scale bar: 20 μm.

To characterize the morphologies and putative synaptic connectivity of the V3-INs, we examined the 3-D morphology of individual V3-INs using confocal microscopy (Fig. 3). The density of DsRed fluorescence is too high in the *Tg(vglut2a:DsRed)^nns9^* transgenic line to accurately trace the processes of a single neuron, so we expressed a photoconvertible protein (Kaede) in *Tg(vglut2a:Gal4)^nns20^;Tg(UAS:Kaede)^s1999t^* larvae to identify and photoconvert single V3-INs (Fig. 3*A*,*B*). Following photoconversion, it was possible to obtain morphologic images of the V3-INs (*n *=* *5 neurons, 4 larvae; Fig. 3*C*). Based on this sample of V3 morphologies, it was evident that variation in the projection patterns of the V3-INs was present. For example, the V3-IN imaged in S10 had minimal projection density dorsal or rostral to the cell body, while the V3-IN in S18 predominantly projected rostrally and dorsally (Fig. 3*C*, lateral projection). Despite these differences, there were some common features of this sample of V3-IN morphologies. First, the imaged V3-INs possessed bilateral projections, but had a higher density of processes ipsilateral to the cell body (Fig. 3*C*, horizontal projection). Second, V3-INs had the greatest density of processes close to the cell body and longer caudal projections than rostral projections (Fig. 3*C*). Third, while the full extent of the descending processes of the S9 and S14 neurons is not presented in Figure 3*C*, V3-INs had a maximum detectable projection distance shorter than 300 μm. The limited projection length we observed may be because of the Kaede photoconversion method used and should be considered a lower bound on the full extent of the projections of the V3-INs. The projection pattern of the V3-INs appeared to innervate the ventral spinal cord, which is populated by MNs, V2a-INs (Eklöf-Ljunggren et al., 2012; Menelaou and McLean, 2013), other V3-INs, KA neurons, and serotonergic neurons (McLean and Fetcho, 2004; Yang et al., 2010; Montgomery et al., 2018).

### The majority of V3-INs are active during spontaneous fictive swimming

**Figure 4.**
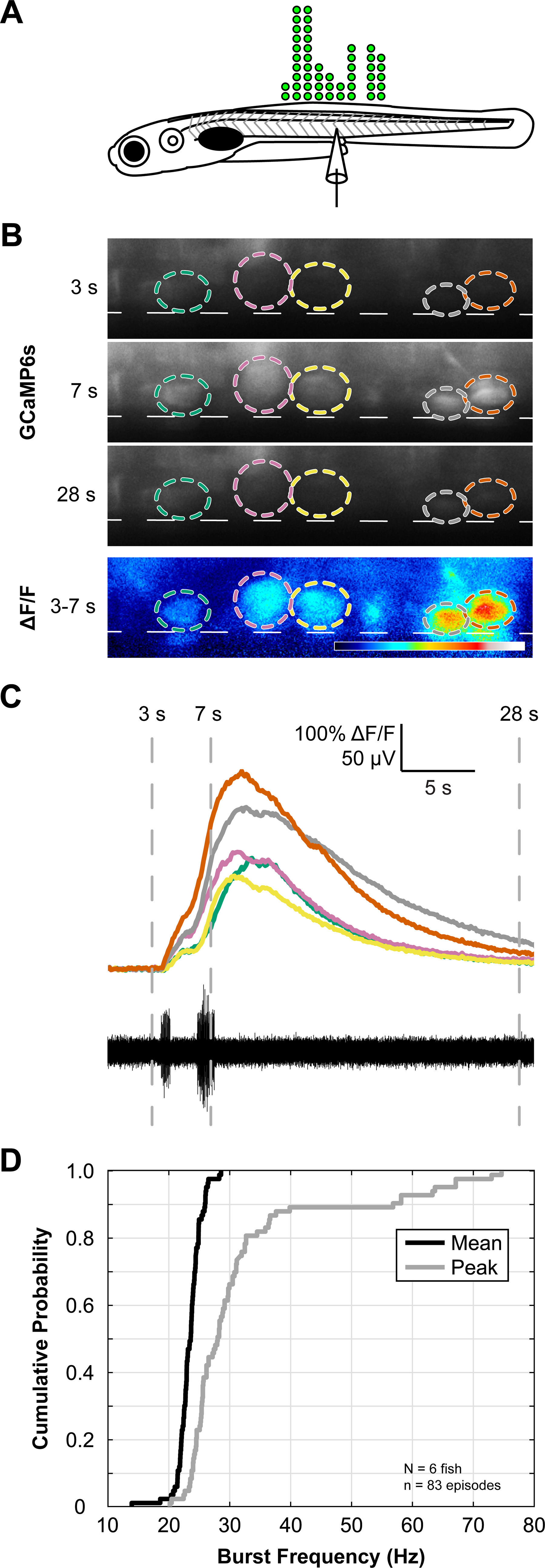
V3-IN activity is temporally correlated to fictive locomotion. ***A***, Schematic representing the number and distribution of V3-INs (green circles) along the rostrocaudal axis of the spinal cord monitored during calcium imaging experiments. ***B***, Each V3-IN in the field of view in Tg(*vglut2a:Gal4ff*)*^nns20^*;Tg(*UAS:GCaMP6s*)*^nk13a^* double transgenic larvae was assigned a ROI during spontaneous fictive swimming. Panels in ***B*** show raw GCaMP6s fluorescence (F) and correspond to numbered time points in ***C***. Bottom panel in ***B*** shows calculated ΔF/F between 3- and 7-s time points. Dashed lines represent ventral boundary of the spinal cord. ***C***, Synchronized recordings of GCaMP6s fluorescence (top) and fictive swimming (bottom) allowed identification of swimming-related neuronal activity. ***D***, Plots of cumulative probability distributions for Mean (black line) and Peak (gray line) burst frequencies.

In mice, there is only indirect evidence supporting temporal correlation between V3-IN activity and locomotion (Borowska et al., 2013). To test the hypothesis that activity in the V3-INs is appropriately timed to provide excitatory drive to MNs during locomotion, we monitored the activity of the V3-INs during spontaneous fictive swimming using a genetically encoded calcium indicator (GCaMP6s; Fig. 4). The activity of V3-INs and fictive locomotor output during spontaneous swimming was measured via simultaneous calcium imaging and PN recording using 4–6 dpf *Tg(vglut2a:Gal4ff)^nns20^;Tg(UAS:GCaMP6s)^nk13a^* larvae (Fig. 4*B*,*C*). We found that the majority, 44 of 48 (91.7%), of V3-INs were active (ΔF/F > 10%) during spontaneous fictive swimming episodes across a range of burst frequencies [mean burst frequency = 23.4 (SD 2.0) Hz, range = 13.9–28.6 Hz; mean peak burst frequency = 31.8 (SD 12.3) Hz, range = 20.2–74.6 Hz; *n *=* *6 larvae, 14 trials, 81 swim episodes; Fig. 4*D*]. Therefore, this provides direct evidence that V3-INs are active during fictive locomotion, which supports the participation of V3-INs in producing locomotor activity.

### Ablation of V3-INs does not affect burst frequency during spontaneous fictive swimming

**Figure 5.**
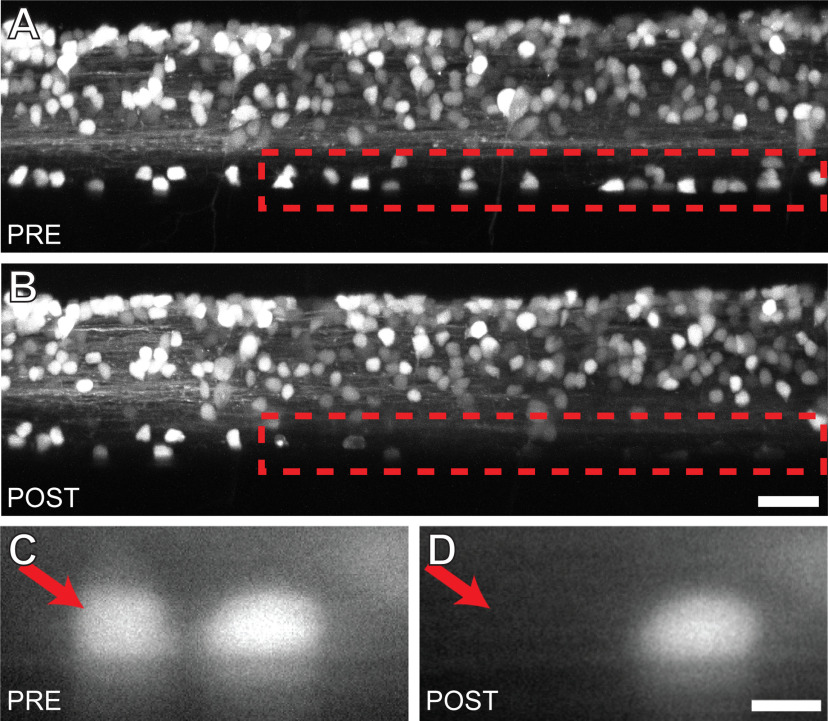
Targeted ablation of V3-INs is both efficient and spatially restricted. ***A***, ***B***, Confocal stacks of the same Tg(*vglut2a:DsRed*)*^nns9^* zebrafish larva before (***A***) and 12 h after (***B***) laser ablation of the V3-INs in the region indicated (red dashed lines). ***C***, ***D***, A magnified view of two V3-INs before (left) and after (right) a single cell laser ablation. Scale bars: 20 μm (***A***, ***B***) and 5 μm (***C***, ***D***).

The previous experiments demonstrated that V3-INs are positioned appropriately and have neurotransmitter phenotype and activity consistent with providing excitatory drive to MNs; however, demonstration of the function of the V3-INs or their necessity for behavior has not been described. To determine whether burst frequency is regulated by V3-IN activity, we performed laser ablation of a large percentage of the V3-IN population (>95%; ∼140 V3-identified somas across body S5–S20; Fig. 5). Three experimental groups were used. (1) The V3 ablation group, in which all V3-INs on both sides of the spinal cord between S5 and S20 were laser ablated (*n *=* *6 larvae; 145 (SD 9.6) neurons per larva). Representative examples of targeted laser ablation efficacy within a restricted region (Fig. 5*A*,*B*) and spatial localization (Fig. 5*C*,*D*) are shown. (2) The sham ablation group, in which laser pulses were delivered 5–20 μm rostral to each identified V3-IN cell body (*n *=* *6 larvae; 130 (SD 10.8) sham targets per larva). (3) The unmanipulated control group was not embedded or exposed to laser pulses (*n *=* *6 larvae).

To measure the effects of V3-IN ablation on burst frequency during spontaneous fictive locomotion, fictive motor activity was measured using extracellular PN recordings 24 h after the ablation procedure. This delay between ablation and recording was necessary to confirm cell death by disambiguating the temporal properties of the loss of fluorescence caused by application of laser pulses. Specifically, laser pulses applied to targeted neurons that does not result in cell death will produce a temporary loss of fluorescence (photobleaching), which will recover since protein synthesis in the viable cells will produce additional fluorescent molecules. In contrast, persistent loss of fluorescence is consistent with absence of new protein synthesis and, thus, cell death.

**Figure 6.**
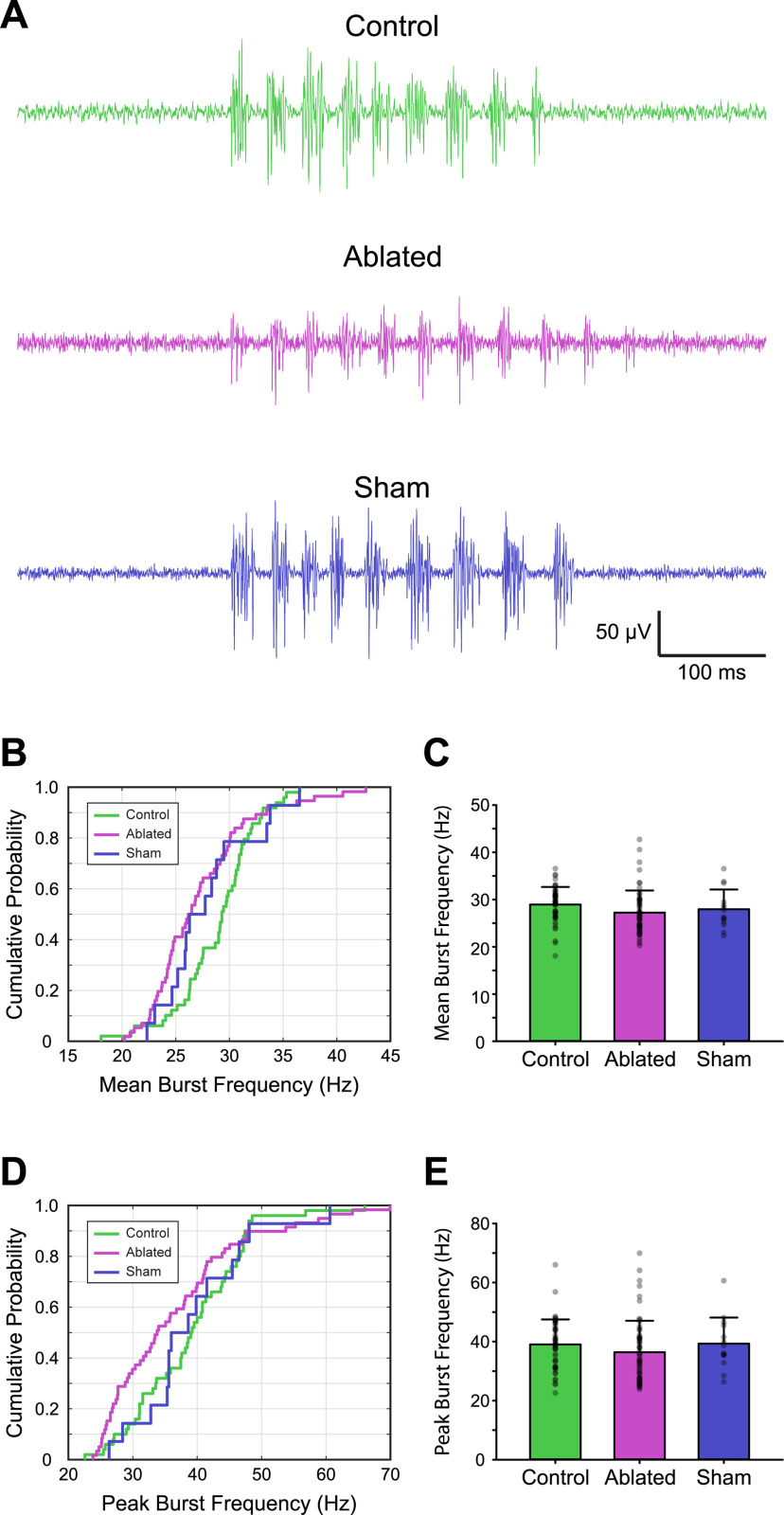
Targeted ablation of V3-INs does not affect burst frequency during spontaneous fictive locomotion. ***A***, Representative traces of extracellular peripheral motor nerve recordings from Control (top), Ablated (middle), and Sham (bottom) groups. Summary plots of cumulative probability distribution (***B***) and mean burst frequencies (***C***). Summary plots of cumulative probability distribution (***D***) and peak burst frequencies (***E***).

Assessment of the mean burst frequency probability distributions showed that the Ablated group was significantly reduced compared with the Control group (Kolmogorov–Smirnov test, D = 0.34, *p* < 0.004; Fig. 6*B*). However, mean burst frequency was not significantly different between either the Control and Sham groups (Kolmogorov–Smirnov test, D = 0.33, *p* = 0.16; Fig. 6*B*) or the Sham and Ablated groups (Kolmogorov–Smirnov test, D = 0.21, *p* = 0.63; Fig. 6*B*). Further, analysis of variance showed that mean burst frequency was not significantly different between Control [29.0 (SD 3.7) Hz], Sham [28.0 (SD 4.2) Hz], and Ablated [27.2 (SD 4.7) Hz] groups (one-way ANOVA, *F* = 2.2, *p* = 0.2; Fig. 6*C*).

Assessment of the peak burst frequency probability distributions showed that there were no significant differences between Control, Sham, and Ablation groups (Kolmogorov–Smirnov test, D < 0.14, *p* > 0.09 for all group comparisons; Fig. 6*D*). This was consistent with an analysis of variance that showed peak burst frequency was not significantly different between Control [39.0 (SD 8.5) Hz], Sham [39.3 (SD 8.8) Hz], and Ablated [36.4 (SD 10.7) Hz] groups (one-way ANOVA, *F* = 1.2, *p* = 0.3; Fig. 6*E*).

Finally, an effect on the coordination of rostrocaudal (ipsilateral) and side-to-side (contralateral) locomotor activity was not observed (data not shown). These results indicate that the population of spinal V3-INs do not play a significant role in establishing burst frequency (a proxy for tail-beat frequency) or coordination during fictive locomotion in larval zebrafish.

### Ablation of V3-INs reduces the proportion of active MNs during spontaneous fictive swimming

**Figure 7.**
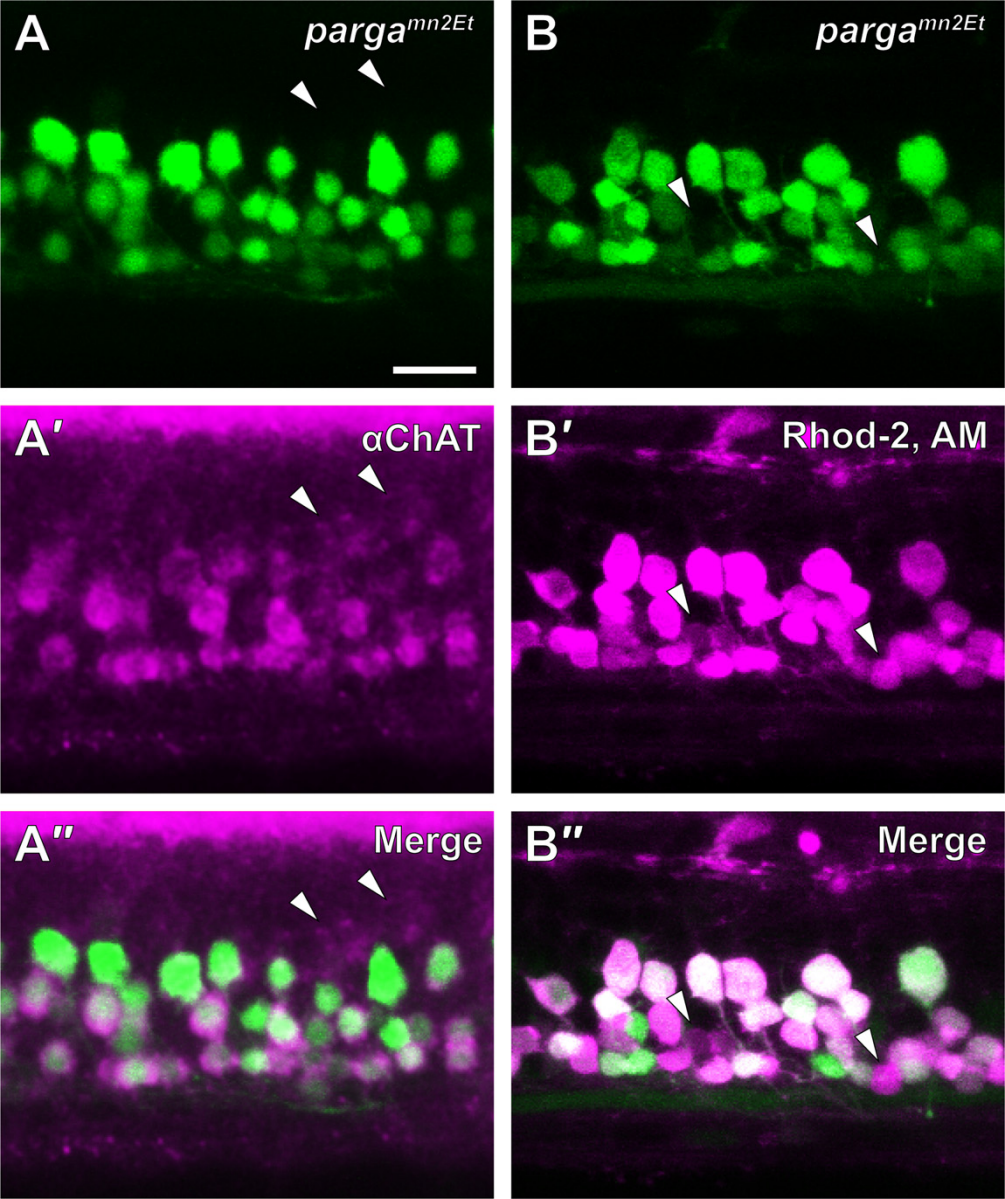
Spinal neurons loaded with rhodamine-2 AM dye are definitively identified as MNs. ***A***, The EGFP-expressing *parga^mn2Et^* enhancer trap line (***A***, green) was labeled with antibodies to choline acetyl transferase (ChAT; ***A′***, magenta). Colabeling (***A′′***) of EGFP with ChAT antibodies indicated that EGFP expression in the *parga^mn2Et^* line is a marker of spinal MNs. White arrowheads indicate ChAT-positive cells that do not contain EGFP (non-MNs). ***B***, Live *parga^mn2Et^* larvae were anesthetized, skinned, and incubated in rhodamine-2 AM dye (Rhod-2, AM). EGFP expression (***B***, green) and Rhod-2, AM labeling (***B′***, magenta), showed that Rhod-2, AM specifically labeled all MNs (***B′′***). White arrowheads indicate two Rhod-2, AM-labeled cells that did not contain EGFP. Scale bar: 20 μm.

Since V3-IN soma position (Fig. 2) and morphologic properties (Fig. 3) suggest that they project to the motor column in the spinal cord and targeted ablation of spinal V3-INs in zebrafish larvae did not affect burst frequency (Fig. 6), we tested the hypothesis that ablation of V3-INs decreases the number of active MNs during spontaneous fictive swimming. To ablate V3-INs without also photo-damaging or bleaching MNs, we used a two-step neuronal targeting strategy. First, V3-INs were identified and ablated using the *Tg(vglut2a:DsRed) ^nns9^* transgenic line. Following recovery from laser ablation, MNs were loaded with a cell-permanent calcium indicator, Calcium Green-1 AM. To verify that spinal neurons loaded with AM dye (Calcium Green-1 and/or Rhod-2) were MNs, we first confirmed motor neuronal specificity of GFP expression in the *parga^mn2Et^* enhancer trap line (Balciunas et al., 2004). *Parga^mn2Et^* larvae immunolabeled for the cholinergic marker ChAT revealed that most GFP-expressing neurons co-labeled with ChAT antibodies [*n *=* *5; 91.3% (SD 2.1); Fig. 7*A*,*A′′*]. A small percentage of dorsally-positioned ChAT+ cells did not colocalize with GFP [4.1% (SD 2.8); Fig. 7*A*,*A′′*, arrowheads); these cells were considered members of a subset of cholinergic spinal interneurons that modulate MN excitability recently identified in zebrafish (Bertuzzi and Ampatzis, 2018). Thus, GFP expression in the *parga^mn2Et^* enhancer trap line was specific to MNs.

Next, to confirm that loading of AM dye was restricted to MNs, the skin was removed from both sides of the body from *parga^mn2Et^* larvae, in which GFP expression was shown to be limited to MNs (Fig. 7*A*,*A′′*), and MNs were labeled with Rhod-2 AM dye (*n *=* *4; Fig. 7*B*,*B′′*). Rhod-2 AM, which was not used for calcium imaging experiments because it did not produce a strong calcium signal, was used as a proxy for Calcium Green-1 AM since the excitation/emission wavelengths of Calcium Green-1 AM overlapped with and could not be disambiguated from GFP expressed in *parga^mn2Et^* MNs. The majority of GFP-expressing MNs co-labeled with Rhod-2 AM [84.3% (SD 4.3); Fig. 7*B*,*B*′′]. A small percentage of GFP-negative cells contained Rhod-2 AM [5.4% (SD 0.5); Fig. 7*B*,*B*′′, arrowheads]. Taken together, these results confirmed that MNs were selectively labeled with AM dye and, therefore, the cell permeable calcium indicator (Calcium Green-1 AM) could be used to monitor *in vivo* calcium activity in spinal MNs.

**Figure 8.**
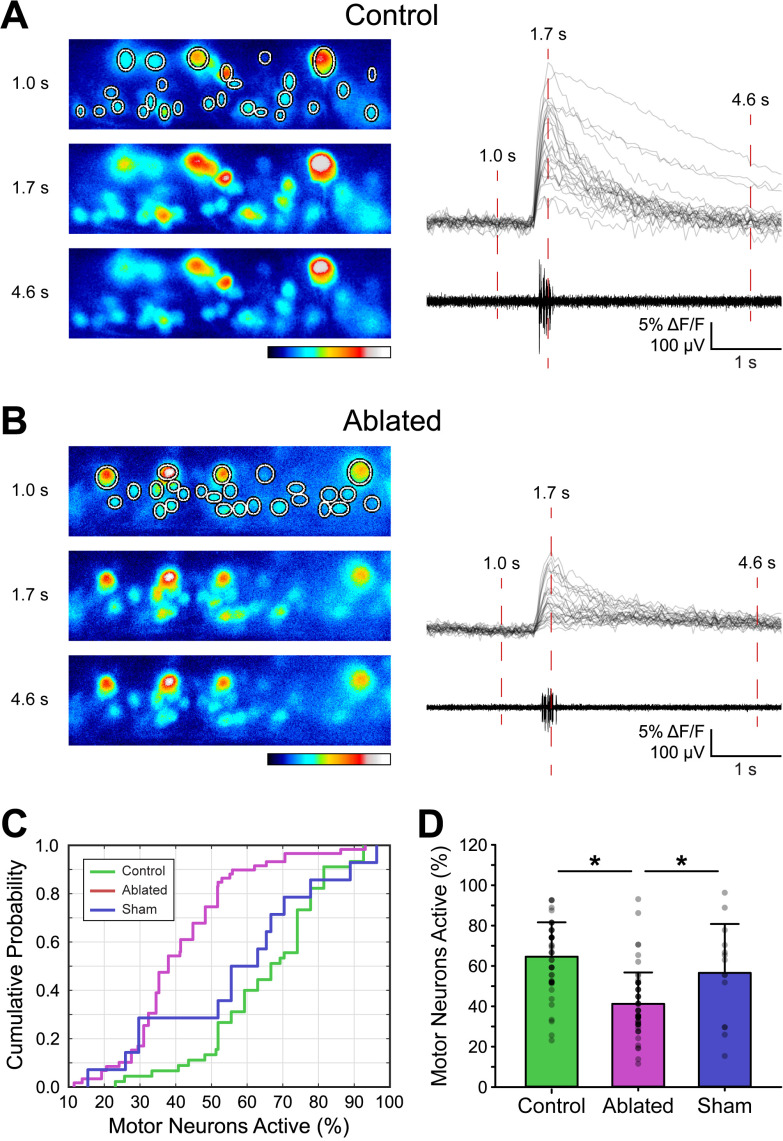
V3-IN laser ablation reduces the proportion of MNs that are active during fictive swimming. MNs in *Tg(vglut2a:DsRed)^nns9^* larvae were loaded with Calcium Green-1 AM dye and assigned ROIs during recording. Synchronous MN fluorescence and fictive swimming recordings were compared between Control, V3-IN Ablated, and Sham ablated preparations. ***A***, ***B***, Pseudocolored Calcium Green-1 AM fluorescence panels correspond to numbered time points in aligned ΔF/F (each gray line represents a MN) and PN traces in a Control preparation (***A***) and a V3-IN Ablated preparation (***B***). Color indicates fluorescence intensity. ***C***, ***D***, Cumulative probability distributions (***C***) and percent of MNs active (***D***) during fictive swimming. Asterisks indicate significant differences at *p* < than 0.01. Error bars represent SD.

To examine the effects of V3-IN ablation on the proportion of spinal MNs active during spontaneous fictive swimming episodes, MN activity in *Tg(vglut2a:DsRed)^nns9^* larvae was measured using *in vivo* calcium imaging by loading MNs with Calcium Green-1 AM, while fictive locomotor activity was measured using extracellular PN recordings (Fig. 8*A*,*B*). We quantified the proportion of active MNs during spontaneous fictive swimming for Control (50 swim episodes, four larvae), Sham Ablated (14 swim episodes, two larvae), and V3-IN Ablated (59 swim episodes, three larvae) groups.

Assessment of the probability distributions showed that the proportion of active MNs in the Ablated group was significantly reduced compared with both the Control (Kolmogorov–Smirnov test, D = 0.68, *p* < 0.001) and Sham (Kolmogorov–Smirnov test, D = 0.54, *p* = 0.001) groups (Fig. 8*C*). The proportion of active MNs was not significantly different between the Control and Sham groups (Kolmogorov–Smirnov test, D = 0.24, *p* = 0.50; Fig. 8*C*). Further, V3-IN ablation had a main effect of significantly reducing the proportion of active MNs during spontaneous fictive swimming [Control mean = 64.6% (SD 17.0); Sham mean = 56.6% (SD 24.2); Ablated mean = 41.2% (SD 15.6); one-way ANOVA, *F* = 25.3; *p* < 0.001; Fig. 8*D*]. Pairwise multiple comparisons (Holm–Sidak; Fig. 8*D*) revealed significant differences for the proportion of active MNs between both the Control and Ablated groups (*t* = 7.02, *p* < 0.001) and the Sham and Ablated groups (*t* = 2.98, *p* = 0.007), but significant differences were not found between Control and Sham groups (*t* = 1.54, *p* = 0.10). These results indicate that V3-INs may participate in regulating locomotor amplitude by recruitment of spinal MNs during spontaneous fictive locomotion.

## Discussion

The goal of this study was to characterize the potential role of V3-INs in regulating locomotor frequency, amplitude, or both, in genetically tractable and optically accessible larval zebrafish. Locomotor amplitude is determined by the recruitment of motor units appropriate to the desired movement: small units for fine behaviors and large units for large movements (Zajac and Faden, 1985; Mendell, 2005). This recruitment pattern of small to large motor units, the so-called size principle, is well established in both limbed (mammalian; terrestrial locomotion) and nonlimbed (swimming) locomotor systems (Henneman et al., 1965; Gustafsson and Pinter, 1984a,b; Cope and Pinter, 1995; McLean et al., 2007; Gabriel et al., 2011). However, investigations into the premotor neural mechanisms that contribute to the regulation of locomotor amplitude have been difficult in mammals for two primary reasons. First, mammalian spinal locomotor circuits are complicated by the requirements to control and coordinate limbs and joints, rather than simply produce a smooth gradient of motion intensity (Grillner, 2003; Grillner and El Manira, 2020). Second, despite recent progress in tool development, *in vivo* functional manipulations of mammalian spinal locomotor circuits using mini-scopes and fiber-optics are both challenging and limited for long-term recordings in freely locomoting animals (Montgomery et al., 2015; Samineni et al., 2017; Canales et al., 2018). Zebrafish larvae provide several advantages over mammalian models for *in vivo* studies: the spinal cords of fish are less complicated than mammals, zebrafish are recognized as a powerful neurogenetic model system, and the translucent nature of zebrafish larvae makes them particularly appropriate for *in vivo* optical methods of investigation. In this study, we found that, unlike the mammalian spinal circuit, V3-INs in the larval zebrafish spinal cord are contained within a single, ventral anatomic domain (Figs. 1, 2) and are not necessary to produce the fictive locomotor rhythm (Fig. 6). Instead, zebrafish V3-INs are swim-active neurons (Fig. 4) that contribute to the recruitment of MNs during fictive locomotion (Fig. 8), and therefore likely participate in the recruitment of motor units during free-moving locomotion.

### V3-INs are a population of previously uncharacterized ventral vglut2a+ neurons in larval zebrafish

Here, we report that almost all ventrally-located neurons labeled by the *Tg(vglut2a:DsRed)^nns9^* and *Tg(vglut2a:Gal4ff)^nns20^* transgenic lines are considered V3-INs (Figs. 1, 2). Since there are no available or published transgenic zebrafish lines that specifically label V3-INs (e.g., *sim1a*-expressing neurons) in larval zebrafish, we recognize that definitive identification of the spinal V3-INs reported here is indirect (Fig. 1). However, we are confident that this approach correctly identifies spinal V3-INs based on the convergence of characteristics, including co-expression of *vglut2a* and the p3 progenitor cell marker *nkx2.2a* (Briscoe et al., 2000; Schäfer et al., 2007), soma position of V3-INs in the p3 domain ventral to the central canal (Figs. 1, 2), and V3-IN neurotransmitter phenotype indicated by the overlap of *vglut2a* and *sim1a* expression in the zebrafish ventral spinal cord (Yang et al., 2010).

The V3-INs, a numerous ventromedial population, are distributed across the rostrocaudal axis of the spinal cord (Fig. 2). It is unlikely that the glutamatergic V3-INs include other identified ventrally-located spinal neurons, such as the GABAergic Kolmer–Agduhr cells (Dale et al., 1987; Bernhardt et al., 1992; Montgomery et al., 2016) or the ventral serotonergic neurons (Van Raamsdonk et al., 1996; Brustein et al., 2003; McLean and Fetcho, 2004; Montgomery et al., 2018). The observed diversity of cell morphology in the V3-IN cell population in the larval zebrafish spinal cord (Fig. 3) is similar to the diversity of V3-INs in the mammalian spinal cord (Borowska et al., 2013; Deska-Gauthier et al., 2020). In mammals, the morphology of V3-IN subtypes is correlated with their circuit function (Borowska et al., 2013). However, we have not found evidence for differential recruitment of V3-INs during fictive swimming at various frequencies (Fig. 4*D*), which would indicate potential functional heterogeneity. It may be that in larval zebrafish V3-INs are a primitive class of excitatory spinal interneurons, as has been previously proposed for *Engrailed1-*expressing inhibitory neurons in zebrafish (Higashijima et al., 2004). However, analysis of cell morphology in zebrafish larvae is complicated by ongoing development in the nervous system and the rostrocaudal developmental gradient (Cole and Ross, 2001). Additional experiments are necessary to clarify whether different morphologies represent distinct functional subtypes of V3-INs, developmental stages of a single morphologic family, or regional specializations of the V3-INs.

### Zebrafish V3-INs are active during fictive locomotion

Results indicating that V3-IN activity is directly correlated to fictive locomotor activity in vertebrate preparations has not been convincingly presented. In mice, there is indirect evidence indicating temporal correlation between V3-IN activity and locomotion (Borowska et al., 2013). Through simultaneous optical and electrophysiological recordings, we showed that activity in most V3-INs is correlated to fictive swimming (Fig. 4) and is therefore appropriately timed to provide excitatory drive to MNs during locomotion. Thus, this is the first direct evidence in any vertebrate preparation that V3-INs are active during fictive locomotion, which provides supporting evidence that they may participate in locomotor activity.

### V3-in activity is necessary for MN recruitment, but not necessary for rhythm generation

Given that V3-INs have been shown to affect the regularity and coordination of locomotor activity in mice (Zhang et al., 2008), it was surprising that laser ablation of V3-INs in larval zebrafish did not affect burst frequency during spontaneous fictive swimming in larval zebrafish (Fig. 6). One interpretation of these findings is that despite similarities in gene expression and neurotransmitter profile, premotor interneuron function is divergent between axial and tetrapod locomotion. The latter argument has been advanced regarding the V2a-INs (Dougherty and Kiehn, 2010; Dougherty et al., 2013); the different effects (or lack thereof) of V3-IN ablation in zebrafish and mice may be a second example of the trend. An important difference between the current study and mouse knock-out experiments is the mechanism of ablation. In the current study, V3-INs were ablated acutely, and little time (∼24 h) was given for circuit compensation. In the mouse experiments, on the other hand, V3-INs were ablated or silenced chronically (Zhang et al., 2008; Rabe Bernhardt et al., 2012), leaving opportunity for physiological compensation. Acute silencing experiments using optogenetics could be performed in both species (Arrenberg et al., 2009; Hägglund et al., 2013), and may demonstrate that this difference between species is because of the experimental manipulation rather than the underlying neural circuit.

Finally, a subset of identified V2a excitatory spinal interneurons has been shown to control the speed of locomotion in larval and juvenile/adult zebrafish (Ausborn et al., 2012; Ampatzis et al., 2014). Of particular interest, recent studies also ascribed a role for regulating locomotor amplitude/vigor to a morphologically and functionally distinct subset of the V2a-INs (Type II, nonbursting) in larval and adult zebrafish (Song et al., 2018; Menelaou and McLean, 2019). Taken together, these results indicate that the general population of V2a-INs is involved in regulating both locomotor speed and amplitude. Importantly, a direct role of V2a-INs in swimming behavior *in vivo* was not shown in either study. To date, however, a population of spinal interneurons that selectively regulates locomotor amplitude has not been identified. Our work provides evidence that, in zebrafish larvae, V3-INs do not contribute to locomotor speed (burst frequency). Specifically, we show that targeted ablation of V3-INs (Fig. 5) reduces the proportion of active MNs during fictive swimming (Fig. 8) but does not affect the range of locomotor frequencies produced (Fig. 6). Thus, we propose that V3-INs are a source of excitation in the vertebrate locomotor neural circuitry that may regulate the amplitude of locomotor output independently of locomotor frequency.

In conclusion, the work presented here addressed an important concept regarding motor control; specifically, a limited understanding of the cellular properties that produce locomotor amplitude in vertebrates. We showed, directly for the first time, that V3-INs in zebrafish larvae are active during *in vivo* fictive locomotion (Fig. 4) and contribute to MN recruitment (Fig. 8). Importantly, targeted ablation of the V3-IN spinal cord population does not affect locomotor frequency (speed; Fig. 6), which suggests a role in motor control rather than rhythm generation. Thus, we propose that the V3-IN population may regulate locomotor amplitude independently of frequency, which is an important functional difference between the V3-INs and the general population of V2a-INs since the latter regulate both frequency and amplitude (Ausborn et al., 2012; Ampatzis et al., 2014; Song et al., 2018; Menelaou and McLean, 2019).

## Synthesis

Reviewing Editor: Silvia Pagliardini, University of Alberta Decisions are customarily a result of the Reviewing Editor and the peer reviewers coming together and discussing their recommendations until a consensus is reached. When revisions are invited, a fact-based synthesis statement explaining their decision and outlining what is needed to prepare a revision will be listed below. The following reviewer(s) agreed to reveal their identity: NONE.

Summary

Using a zebrafish model, the authors examine the role of V3 interneurons in swimming using a combination of several transgenic fish lines, population mapping, calcium imaging, and acute neuronal ablation. The authors demonstrate that, based on location, V3 INs may comprise a single population. The V3 INs are active during locomotion and V3 IN ablation affects recruitment of motor neurons but not locomotor frequency. This demonstrates the role of a specific population of spinal interneurons in swimming. Comparisons with data from genetic ablation of the analogous population in the mouse may provide further insight into the control of locomotion in both animals.

The study has many strengths. The manuscript is well-written, follows logically, and the figures are clear. The use of laser ablation has many advantages over chronic genetic manipulation. Frequency and amplitude are expected to have at least some degree of independent control but none of the canonical populations had been identified to be involved exclusively in amplitude regulation to date. Therefore, this will be of interest to the field as it both further reveals genetically identified locomotor circuits in zebrafish and provides additional fodder for comparative studies and inferences to mammals.

The weaknesses were relatively minor and are many related to lacking information that limits the interpretation of the data as detailed below:

1. Data demonstrating that the activity of V3 INs correlates with swimming is convincing. It is not clear as to whether the V3 INs are providing a tonic level of activation during swimming to bring the motor neurons closer to threshold or if this is a phasic (on cycle) activation related to CPG and/or descending drive. If the population is rather homogenous, given that >95% of the population is active during swimming, a small number of intracellular recordings has the potential to complement the imaging data. This could provide useful information and would demonstrate whether V3 interneurons are rhythmically active, their activity at different frequencies, and would allow the authors to compare their activity with other neuronal types.

2. Some key data are presented as means where individual data points should be shown, particularly those which support major conclusions of the study (i.e. data in Table 1, although no differences, they are essential to the conclusions, as are the proportion of motor neurons active).

3.There is no mention of where the V3 neurons are projecting to and although this will not alter the main conclusions of the study (unless separate populations are found based on projection), this information would enhance the interpretation of the results. In the mouse, ventral V3 INs project contralaterally, ipsilaterally to more lateral V3s, and directly to ipsilateral motor neurons. Are the zebrafish V3 INs a subset of these, recruiting motor neurons on the same or opposite sides?

4. Is it possible to selectively stimulate these interneurons using optogenetics to determine if they

provide direct excitatory drive to motoneurons? This is implicitly assumed but it is not shown in this study.

5. It would also be informative to show behavioral analysis of the freely swimming larvae following V3 ablation. The amplitude and frequency of the tail beat, the swimming velocity and distance travelled could be analyzed. This will allow the authors to link the changes in the activity of motoneurons to swimming behavior in vivo. Indeed, there are no clear effects on fictive swimming following V3 ablation (presumably the amplitude of swim bursts is hard to compare across animals?), yet the reduction in MN activity would be expected to have an effect that may be captured more clearly using behavioral analysis.

6.Discussion should mention out some of the limitations of this study. In particular, the lack of a definitive identification of V3 interneurons and the reliance on an indirect approach for determining their putative identity.

7. Why were the ablated and sham animals not tested the same day? It would be useful to see if the effects seen are similar or different if the animals are tested the same day vs the following day.

Minor

- Lines 43, 55, 504-5: V2a INs have been associated with locomotor frequency in zebrafish but not the mouse. The mouse references listed show that increasing numbers of V2a INs become suprathreshold at increasing speeds and they are essential for left/right alternation at relatively high speeds; but not that they are involved in frequency per se.

- Lines 462-3: Recruitment V3 INs at various frequencies is mentioned but it is not clear from the experiments presented that evidence of V3 recruitment at different frequencies was assessed. The proportion of active V3 neurons was very high but the frequency range of the spontaneous episodes was not given for this part of the study. (It is included for the MN imaging in Fig 6.)

-Typo in Figure 1- nxk2.2a:mEGFP should be changed to nkx2.2a:mEGFP

- Fig 3 legend - The lettering in the second half of the legend does not match the figure.

## Author Response

Dear Reviewers,

We appreciate your thoughtful comments and insights about our manuscript entitled “V3 Interneurons are Active and Recruit Spinal Motor Neurons During In Vivo Fictive Swimming in Larval Zebrafish” (eNeuro eN-NWR-0476-21). Although I fully agree with the important issues identified, I do not believe that it will be possible for us to collect the data from the suggested experiments within the time frame provided (3 months). First, the sample sizes for these (pilot) experiments are currently small, so I am not yet confident in the robustness of the results, and it will take significant effort to collect these data sets. Second, I currently do not have access to the equipment (Digital Micromirror Device) necessary to complete the data collection. Finally, I propose that the current version of the manuscript sets up a solid foundation from which we can extend our studies of the V3 neurons, which would be sufficient for an independent, follow-up manuscript.

Major Comments

1. A small number of intracellular recordings has the potential to complement the imaging data: We agree and have acquired preliminary electrophysiological data (n = 2) indicating that the V3-INs (at least a subset) are rhythmically active during fictive swimming.

2. Individual data points should be shown: As suggested, we have added the individual data points to Figures 6 (new) and 8 (revised).

3. Morphological characterization of V3-INs: We have included a data set (n = 5) to show the basic morphological characteristics of the spinal V3-IN population.

4. Provide evidence for direct excitatory drive from V3-INs to motor neurons: We recently borrowed a Digital Micromirror Device (DMD) to collect preliminary data on the synaptic connectivity between V3 neurons and motor neurons. Using this loaner system, we identified a monosynaptic excitatory synapse from V3-INs to, at least, a subset of motor neurons (n = 2). Unfortunately, I do not currently have access to this or another DMD to complete these experiments.

5. Behavioral analysis of freely swimming larvae following V3 ablation: We agree that this data is critical to show a direct behavioral readout for the role of V3-INs in regulating locomotor amplitude; this is the basis of a revised NIH proposal to be submitted in Cycle I of 2022.

6. Discussion of the limitations of this study: Agreed and addressed in the Discussion (lines 506-16 and 588-99).

7. Why were the ablated and sham animals not tested on the same day: To clarify this issue, we added the following text (lines 390-96): “This delay between ablation and recording was necessary to confirm cell death by disambiguating the temporal properties of the loss of fluorescence caused by application of laser pulses. Specifically, laser pulses applied to targeted neurons that does not result in cell death will produce a temporary loss of fluorescence (photobleaching), which will recover since protein synthesis in the viable cells will produce additional fluorescent molecules. In contrast, persistent loss of fluorescence is consistent with absence of new protein synthesis and, thus, cell death”.

Minor comments

1. V2a INs association with locomotor frequency in zebrafish but not the mouse: Thanks for catching this citation error; it has been corrected.

2. Evidence for V3 recruitment at different frequencies: We added cumulative probability distribution plots for Mean and Peak Burst Frequency in Figure 4, panel D (revised from original Figure 3) to indicate the frequency ranges.

3. Typo in Figure 1: Corrected.

4. Lettering in legend for Figure 3: Corrected.
